# Loss of tumor suppressors KAI1 and p27 identifies a unique subgroup of primary melanoma patients with poor prognosis

**DOI:** 10.18632/oncotarget.4854

**Published:** 2015-07-14

**Authors:** Guohong Zhang, Yabin Cheng, Guangdi Chen, Yun Tang, Gholamreza Ardekani, Anand Rotte, Magdalena Martinka, Kevin McElwee, Xuezhu Xu, Qi Wang, Youwen Zhou

**Affiliations:** ^1^ Department of Dermatology and Skin Science, Vancouver Coastal Health Research Institute, University of British Columbia, Vancouver, British Columbia, Canada; ^2^ Department of Pathology, Shantou University Medical College, Shantou, Guangdong, China; ^3^ Bioelectromagnetics Laboratory, Zhejiang University School of Medicine, Hangzhou, Zhejiang, China; ^4^ Department of Pathology, Vancouver Coastal Health Research Institute, University of British Columbia, Vancouver, British Columbia, Canada; ^5^ Department of Dermatology, 2nd Affiliated Hospital, Dalian Medical University, Dalian, China; ^6^ Dermatologic Oncology Program, British Columbia Cancer Agency, Vancouver, British Columbia, Canada

**Keywords:** primary melanoma, KAI1, p27, prognostic marker, subgroup

## Abstract

Primary melanoma, a highly aggressive malignancy, exhibits heterogeneity in biologic behaviors, clinical characteristics, metastasis potential and mortality. The present study sought to identify the molecular signatures that define a subgroup of primary melanomas with high risks of metastasis and mortality.

First, we identified the markers that best differentiated metastatic melanomas from primary melanomas by examining the expression of seven previously reported biomarkers (BRAF, Dicer, Fbw7, KAI1, MMP2, p27 and Tip60) in a training cohort consisting of 145 primary melanomas and 105 metastatic melanomas. KAI1 and p27, both tumor suppressors, emerged as best candidates. Loss of both tumor suppressors occurred in the majority (74.29%) of metastatic melanomas. Further, a subset (metastatic like, or “ML”, 33.10%) of primary melanomas also lost these two tumor suppressors. Kaplan-Meier analysis indicated that ML subgroup of primary melanoma patients had much worse 5 year survival compared with other primary melanoma patients (*P* = 0.002). The result was confirmed in an independent validation cohort with 92 primary melanomas (*P* = 0.030) and in the combined cohort with 237 melanoma patients (*P* = 3.00E-4). Additionally, compared to KAI1 and p27 as an individual prognostic marker, the combined signature is more closely associated with melanoma patient survival (*P* = 0.025, 0.264 and 0.009, respectively).

In conclusion, loss of both KAI1 and p27 defines a subgroup of primary melanoma patients with poor prognosis. This molecular signature may help in metastatic melanoma diagnosis and may provide information useful in identifying high-risk primary melanoma patients for more intensive clinical surveillance in the future.

## INTRODUCTION

Cutaneous melanoma, arising from abnormal proliferation of melanocytes in the epidermis, is one of the most aggressive forms of skin cancer [1]. Tumor metastasis to distant organs is responsible for the majority of melanoma-related death; only 14% of metastatic melanoma patients survive for 5 years [2]. The treatment of metastatic melanoma has been notably improved by recent development of the specific MAP-Kinase (BRAFV600E, MEK) inhibitors and the immune checkpoint antibodies (anti-CTLA-4, anti-PD1/PDL1) [3-6]. Both therapies have shown survival benefit for patients with metastatic melanoma, however, both regimens have their own limitations [7]. Patients with metastatic melanoma still face a very poor prognosis: with a median survival of well under one year [8].

Melanoma has long been recognized as a highly heterogeneous disease [9-10]. It has been shown that approximately 50% of patients develop metastases within 15 years after treatment of the primary melanoma, and the occurrence rate of metastasis is 15% in patients with a thin (<1 mm) melanoma after their initial treatment [11]. However, the currently used AJCC (American Joint Committee of Cancer) staging system, which is based on clinical and histological parameters, although highly useful as a general guideline for prognostication, cannot precisely define such metastasis/mortality risks in many cases. For example, sentinel node biopsy has been recommended by AJCC as an important prognostic factor in early stage melanoma. But one third of patients diagnosed with metastatic melanoma do not present with regional lymph node involvement, and therefore are not detected by sentinel lymph node biopsy [12]. Some other factors that are not mentioned in AJCC staging system have potential to stratify metastatic risk. For example, a very recent study demonstrated that melanogenesis (the biochemical process to produce melanin by melanocytes) correlates with melanoma clinical outcome and promotes tumor progression [13-15]. Development of a prognostic assay that could triage high risk primary melanomas will be highly valuable for melanoma management and beneficial for melanoma patients.

To metastasize, tumor cells need to gain a series of biological capabilities to achieve invasion, distant growth, extravasation and colonization [16]. Acquisition of these capabilities requires certain genetic and epigenetic events occurring in the tumor cells. Therefore, the heterogeneity of tumors, including metastatic potential, is dictated by underlying genetic and molecular alterations [17]. In this regard, molecular or genetic expression profiles and potential biomarkers could be utilized to evaluate the metastatic risk of primary melanoma and predict patient survival [18-23]. However, previous studies on melanoma indicated that individual biomarkers are of narrow statistical significance and are unlikely to be widely adopted [23], so combinations of two or more biomarkers have gained increasing importance. Gould-Rothberg et al. found a tissue microarray and genetic-algorithm based five-marker prognostic model for stage II melanoma patients [24]; while Meyer et al. demonstrated a seven-marker signature to predict clinical outcome in malignant melanoma [25]. So far, none of these combinative biomarkers have become standard use in the clinical setting. These prognostic models still need to be validated in further prospective tests or clinical trials.

In this present study, we retrieved the melanoma tissue microarray clinical data for seven previously reported independent prognostic biomarkers [26-32], performed statistical analysis, and identified a molecular signature (loss of both KAI1 and p27) that defined a metastatic-like-subgroup (ML) among patients with primary melanomas, in both the training dataset and in a validation dataset. Our data showed that the ML subgroup had a poor 5-year melanoma-specific survival compared to the non-metastatic like (NML) subgroup (*P* < 0.001, Log-rank test). Additionally, multivariate Cox regression analysis revealed that KAI1-/p27- is an independent prognostic factor in primary melanomas, showing a stronger correlation with patient survival than when used as individual markers.

## RESULTS

### KAI1 and p27 differentiate metastatic from primary melanomas

**Figure 1:**
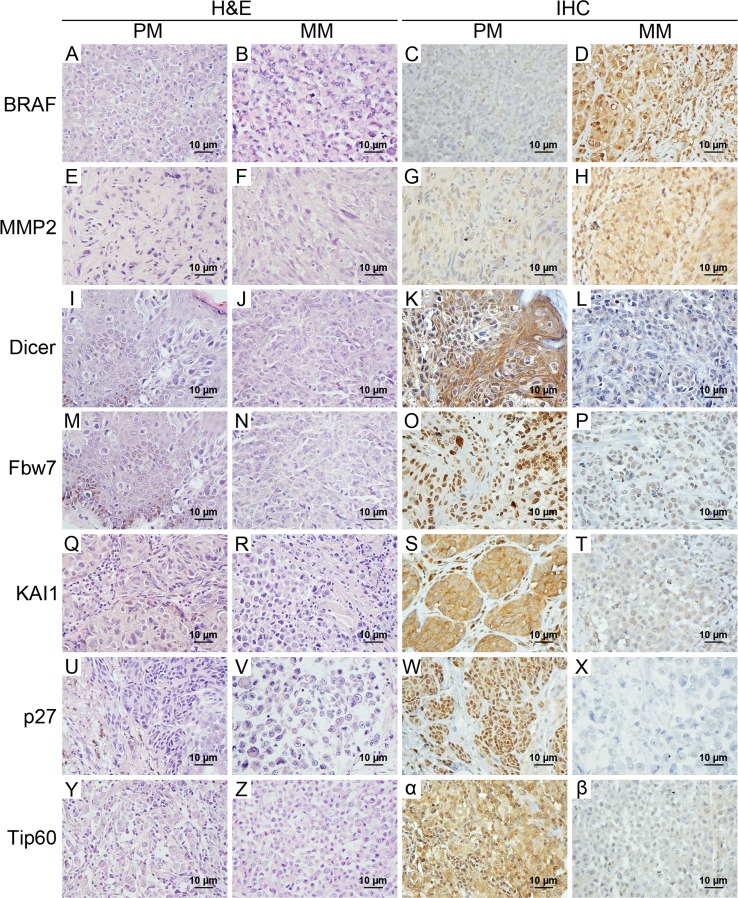
Representative H&E and immunohistochemical staining of 7 candidate biomarkers in primary melanomas and metastatic melanomas Cytoplasmic staining was investigated for BRAF, MMP2, Dicer, KAI1, and Tip60, and nuclear staining was observed for Fbw7 and p27. Metastatic melanomas had overexpression of BRAF and MMP2, but did not express/had low levels of Dicer, Fbw7, KAI1, p27 and Tip60 compared with primary melanomas. Magnification used × 400 for H & E and immunohistochemical staining. H & E, hematoxylin and eosin; IHC, immunohistochemistry; PM, primary melanoma; MM, metastatic melanoma.

First of all, logistic regression analysis was performed to identify the signature proteins that most efficiently discriminated metastatic from primary melanomas. Seven previously reported independent prognostic biomarkers were selected based on the correlation with metastatic tumors [27, 35-40] (Table 1). Representative staining of BRAF, Dicer, Fbw7, KAI1, MMP2, p27 and Tip60 is shown in Figure 1. As shown in the images, cytoplasmic staining was observed for BRAF, MMP2, Dicer, KAI1, and Tip60, whereas nuclear staining was observed for Fbw7 and p27.

**Table 1:** Seven selected candidate biomarkers

Marker	Full name	Function
BRAF	V-raf murine sarcoma viral oncogene homolog B	Proto-oncogene
Dicer	Dicer 1, ribonuclease type III	Endoribonuclease
Fbw7	F-box and WD repeat domain containing 7	E3 ubiquitin protein ligase
KAI1	CD82 molecule	Metastasis suppressor
MMP2	Matrix metallopeptidase 2	Matrix metalloproteinase
P27	Cyclin-Dependent Kinase Inhibitor 1B	Tumor suppressor
Tip60	Tat-interactive protein 60 kDa	Regulates metastasis suppressor

**Figure 2:**
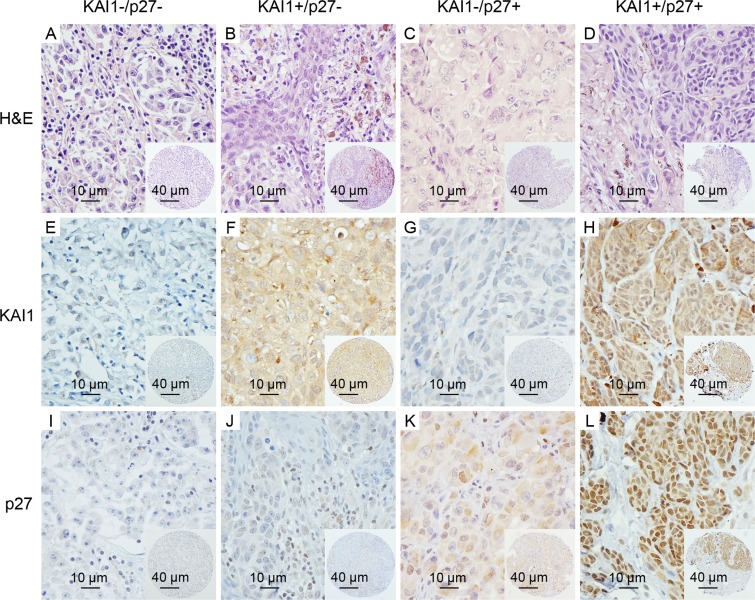
Representative images of KAI1 and p27 staining in subgroups of primary melanomas **A.**-**D.** the H & E staining images of representative cores are shown for each subgroup; **E.** and **I.**, absent/minimal cytoplasmic expression of KAI1 and nuclear expression of p27 (KAI1-/p27-) were seen in 48 of the 145 cases; **F.**-**H.**, **J.**-**L.**, moderate or high cytoplasmic expression of KAI1 or nuclear expression of p27 (KAI1+/p27- or KAI1-/p27+), or expression of both (KAI1+/p27+), are shown in 97 of 145 primary melanoma patients. (magnification: × 400); insets show corresponding tissue microarray cores (magnification: × 100).

Univariate analysis showed that BRAF, Dicer, KAI1, P27 and Tip60 were differentially expressed in metastatic melanoma as compared to primary melanoma. Multiple logistic regression analyses revealed four biomarkers that were significant: BRAF (*P* = 0.037), Tip60 (*P* = 0.029), p27 (P = 9.677E-5) and KAI1 (*P* = 6.321E-5); KAI1 and p27 were the two most significant biomarkers (Table 2). KAI1 and p27 were expressed in 95.24% and 78.10% of metastatic tumors, and in 66.21% and 50.34% of primary melanomas, respectively. This difference between primary melanoma and metastatic melanoma expression was highly significant (*P* < 0.001), indicating that loss of KAI1 or p27 may represent a relatively robust feature of more advanced melanomas. Representative KAI1 and p27 staining patterns in primary melanomas are shown in Figure 2. Furthermore, we ran the same analysis on the combination of KAI1 and p27, and found loss of both proteins better differentiated metastatic from primary melanomas, suggesting that double loss of both KAI1 and p27 can serve as a potential signature for metastatic like (ML) primary melanomas.

**Table 2:** Expression of seven biomarkers in primary versus metastatic melanomas

Biomarker	Primary melanoma No. (%)		Metastatic melanoma No. (%)		*P* value	
	−	+	−	+	Univariate	Multivariate
BRAF	79 (54.48)	66 (45.52)	36 (34.29)	69 (65.71)	0.002	0.037
Dicer	10 (6.90)	135 (93.10)	23 (21.90)	82 (78.10)	0.001	0.053
Fbw7	22 (15.17)	123 (84.83)	21 (20.00)	84 (80.00)	0.318	0.666
KAI1	96 (66.21)	49 (33.79)	100 (95.24)	5 (4.76)	4.000E-8	6.321E-5
MMP2	102 (70.34)	43 (29.66)	63 (60.00)	42 (40.00)	0.089	0.166
P27	73(50.34)	72(49.66)	82(78.10)	23(21.90)	8.130E-6	9.677E-5
Tip60	30(20.69)	115(79.31)	46(43.81)	59(56.19)	8.768E-6	0.029

### The subgroup of primary melanoma with KAI1-/p27- signature had poor survival in the training cohort

**Figure 3:**
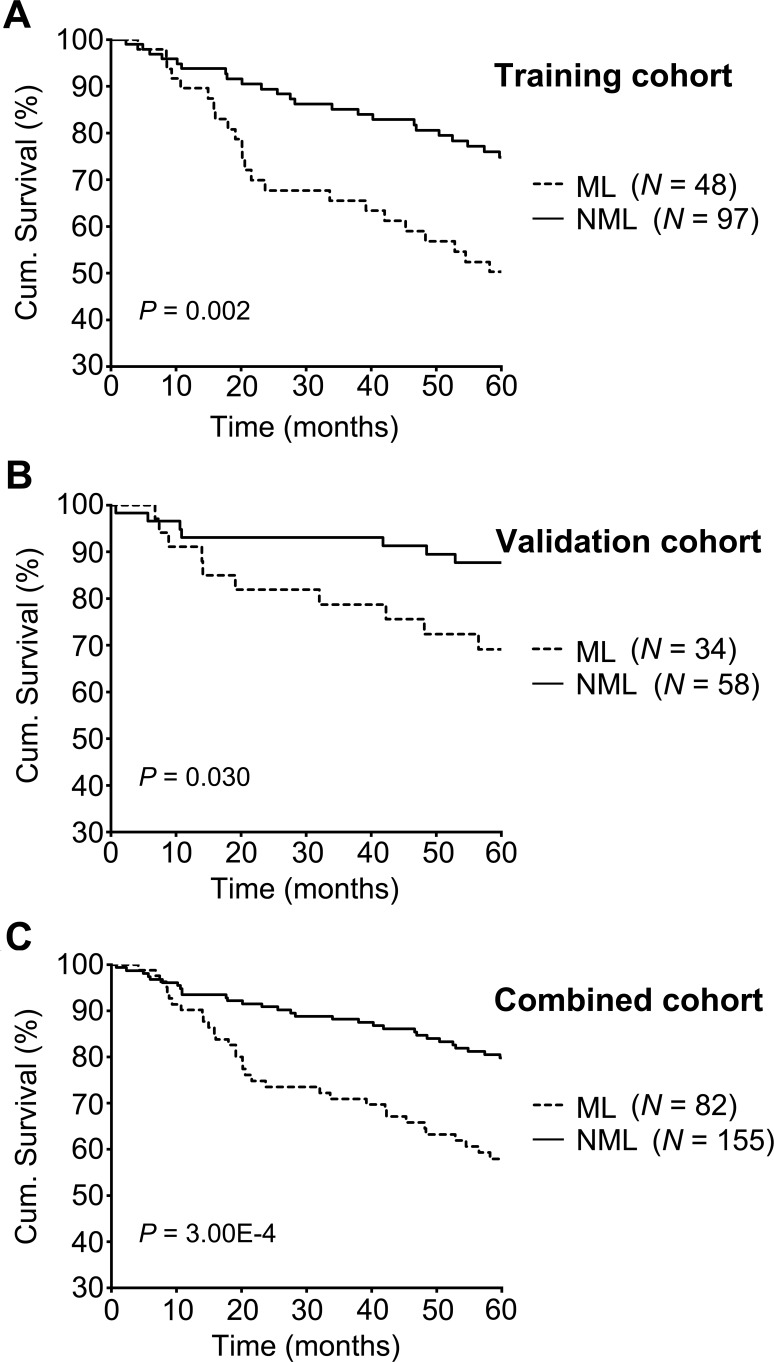
5-year melanoma-specific survival analyses in training, validation and combined cohorts Melanoma-specific survival in different subgroups of primary melanomas was analyzed by the Kaplan-Meier survival model. Patients in the ML subgroup showed poorer prognosis than patients in the NML subgroup in the training cohort **A.** poor survival for patients in the ML subgroup was found in the validation cohort **B.** and combination cohort **C.** Log-rank *P* value is indicated in the graphs.

To evaluate whether a KAI1-/p27- signature can distinguish high risk primary melanomas, and examine the prognostic value of this combined marker, we separated 145 primary melanomas into two subgroups: a metastasis-like group (ML) of 48 patients with negative expression of both KAI1 and p27 (33.10%), and a non-metastasis-like group (NML) of 97 patients with positive expression of either KAI1 or p27 or both (66.90%) (Table 3). Representative images of the expression patterns are shown in Figure 2. Kaplan-Meier survival curves showed that melanoma-specific survival is significantly reduced for patients in the metastasis-like group (52.10%) compared to those in the non-metastasis-like subgroup (76.30%, *P* = 0.002, Figure 3A).

**Table 3:** Subgroups of primary melanoma in training cohort, validation cohort and combined cohort

Population	Subgroup	Expression	Case no. (%)
Training cohort (145 cases)	ML	KAI1-/P27-	48 (33.10)
	NML	Either KAI1 or p27 is negative	97 (66.90)
Validation cohort (92 cases)	ML	KAI1- P27-	34 (37.00)
	NML	Either KAI1 or p27 is negative	58 (63.00)
Combined cohort (237 cases)	ML	KAI1- P27-	82 (34.60)
	NML	Either KAI1 or p27 is negative	155 (65.40)

ML, metastasis-like; NML, non-metastasis-like

### The ML subgroup of primary melanoma had poor survival in both the independent validation cohort and the combined cohort

To validate our results, 92 additional independent primary melanoma patients were classified into NML and ML subgroups and analysed (Table 3). Survival analysis showed that the NML group had significantly better disease-specific survival (87.9% in NML versus 70.6% in ML, *P* = 0.030, Figure 3B). In addition, the same trend was observed in the combined patient's cohort: 79.8% of NML subgroup patients can survive for 5 years, while this number drops to 57.9% in ML subgroup (*P* = 3.00E-4, Figure 3C, Table 3).

**Table 4:** Clinicopathological characteristics of NML and ML subgroups in combination cohort of patients with primary melanoma

Variable	ML (N = 82)	NML (N = 155)	*P* value
Age	63.42 ± 1.97	59.48 ± 1.53	0.123
Sex			0.194
Male	48(58.54)	77(49.68)	
Female	34(41.46)	78(50.32)	
Thickness	4.94 ± 0.73	2.66 ± 0.24	0.001
Ulceration (%)			0.117
Absent	57(69.51)	122(78.71)	
Present	25(30.49)	33(21.29)	
Subtype (%)			0.830
AL	4(4.88)	6(3.87)	0.714
LM	8(9.76)	18(11.61)	0.663
N	20(24.39)	28(18.06)	0.249
SS	27(32.93)	62(40.00)	0.285
Other	7(8.54)	13(8.39)	0.969

### The KAI1-/p27- signature is an independent factor for primary melanoma survival

Univariate Cox proportional hazards regression analysis showed that the ML subgroup had significantly worse disease-specific survival, with a hazard ratio of 2.421 (95% CI: 1.481-3.972; *P* = 4.649E-4) (Table 5), indicating that the risk of dying in the ML subgroup was 2.421 times greater compared to that of the NML subgroup. Multivariate Cox proportional hazards regression model adjusted for age, gender, thickness, ulceration were assessed (Tables 6, 7, 8). In the multivariable Cox model, the KAI1-/p27- signature was found to be of independent prognostic significance for melanoma-specific survival (*P* = 0.009). More important, compared to KAI1 and p27 as an individual prognostic marker, the KAI1-/p27- signature is more closely associated with melanoma patient survival: the *P* value is 0.009 for KAI1-/p27-, compared to 0.025, 0.264 for KAI1 and p27, respectively (Tables 6, 7, 8).

**Table 5:** Univariate Cox proportional hazard regression analysis in combined cohort (237 primary melanoma patients)

	Hazard ratio	95% CI	*P* value
Age: > 60 vs ≤ 60 y	2.372	1.392 - 4.041	0.001
Gender: male vs female	0.980	0.601 - 1.615	0.937
Thickness: > 2 vs ≤ 2 mm	4.883	2.692 - 8.863	1.791E-7
Ulceration: + vs −	4.354	2.652 - 7.168	6.510E-9
KAI1: − vs +	2.996	1.602 - 5.621	0.001
p27: - vs +	1.690	1.020 - 2.815	0.043
KAI1/p27: ML vs NML	2.421	1.481 - 3.972	4.649E-4

CI: confidence interval; ML: metastasis-like subgroup, KAI1−/p27−; NML: non-metastasis-like subgroup: KAI1−/p27+ or KAI1+/p27− or KAI1+/p27+.

**Table 6:** Multivariate Cox proportional hazard regression analysis in combined cohort (237 primary melanoma patients) for KAI1 as a single biomarker

	Hazard Ratio	95% CI	*P* value
Age: > 60 vs ≤ 60 y	1.271	0.727-2.224	0.400
Gender: male vs female	0.985	0.609-1.692	0.954
Thickness: > 2 vs ≤ 2 mm	3.256	1.728-6.137	2.609E-4
Ulceration: + vs -	2.616	1.535-4.459	4.082E-4
KAI1: - vs +	2.083	1.096-3.953	0.025

CI: confidence interval.

**Table 7:** Multivariate Cox proportional hazard regression analysis in combined cohort (237 primary melanoma patients) for p27 as a single biomarker

	Hazard Ratio	95% CI	P value
Age: > 60 vs ≤ 60 y	1.265	0.725-2.205	0.408
Gender: male vs female	0.915	0.550-1.522	0.732
Thickness: > 2 vs ≤ 2 mm	3.114	1.647-5.888	3.158E-4
Ulceration: + vs −	2.900	1.689-4.979	1.129E-4
p27: − vs +	1.345	0.798-2.278	0.264

CI: confidence interval.

**Table 8:** Multivariate Cox proportional hazard regression analysis in combined cohort (237 primary melanoma patients) for combined KAI1-/p27- signature

	Hazard Ratio	95% CI	P value
Age: > 60 vs ≤ 60 y	1.277	0.727-2.244	0.394
Gender: male vs female	0.894	0.523-1.486	0.665
Thickness: > 2 vs ≤ 2 mm	3.114	1.647-5.888	4.757E-4
Ulceration: + vs −	3.005	1.760-5.130	5.525E-5
KAI1/p27: ML vs NML	1.957	1.182-3.236	0.009

CI: confidence interval; ML: metastasis-like subgroup, KAI1-/p27-; NML: non-metastasis-like subgroup: KAI1-/p27+ or KAI1+/p27- or KAI1+/p27+.

### Loss of KAI1 and p27 in primary melanoma was correlated with thickness

It is noteworthy that ML subgroup exhibited a relatively high hazard ratio for 5-year melanoma-specific survival (HR = 1.957; 95% CI = 1.182-3.236), as compared to tumor thickness (HR = 3.114; 95% CI = 1.647-5.888) when analyzed together, suggesting a potential interdependence between KAI1- /p27- signature and tumor thickness. To investigate the clinical phenotype in the ML subgroup of primary melanoma patients, we compared the relevant patient clinicopathologic characteristics and histological features between ML and NML subgroups in the combination cohort. The associations of the KAI1-/p27- signature with other patient and tumor characteristics are shown in Table 4. There was no difference in age, gender, clinical subtype and ulceration between ML and NML subgroups. However, we found that the ML subgroup was characterized by greater mean thickness compared to the NML subgroup (4.94 vs. 2.66 mm; *P* = 0.001, Table 4), suggesting that the ML subgroup has more aggressive characteristics.

## DISCUSSION

Since Fidler first demonstrated the heterogeneity of mouse melanoma cells with respect to metastatic potential in 1973 [41], several genome-wide high-throughput studies have described gene expression signatures to predict metastasis of primary melanoma patients. However, the gene-sets identified have shown minimal overlap between various studies, and lack the convenience and simplicity necessary for clinical application [42].

In the present study, we performed statistical analysis on a patient cohort with large sample size, and identified the metastasis-like subgroup of primary melanomas in order to develop a clinically effective classification model. We demonstrated that simultaneous loss of both KAI1 and p27 was a novel molecular feature associated with metastasis, discriminating between primary and metastatic melanomas, identifying a metastasis like subgroup (ML subgroup) within primary melanoma patients, and constituting a strong prognostic marker for poor survival in patients with primary melanoma.

Initially, our data demonstrated that KAI1- and p27- were the two markers among 7 previously identified independent markers that showed the most significant difference in expression level between metastatic melanomas (95.24% vs. 78.10%) and primary melanomas (66.21% vs. 50.34%, *P* < 10E-4) (Table 2). This result was consistent with the previous finding that highly aggressive tumors showed the lowest KAI1 and p27 expression levels [43-44]. Our study extends the previous work in that the combination of KAI1 and p27 loss is a metastatic feature and significantly differentiates metastatic melanomas from primary melanomas (*P* < 0.001).

Tumor metastasis suppressor KAI1 has previously been shown to interfere with multiple steps of the metastatic cascade, including proliferation, invasion, and migration [45]. Also the inverse association of KAI1 expression with formation of metastasis was reported [46]. Studies from our group have found dramatic decrease of KAI1 protein expression in human melanomas using tissue microarray technology, and KAI1 expression was negatively correlated with patient outcome [32]. The prognostic value and the role of p27 in cancer metastasis have been intensely reported [47-48]. However, no studies have examined the combination of KAI1 and p27, two important proteins for metastatic disease, to identify high-risk primary tumors.

Using the metastatic signature (loss of both KAI1- and p27-), a metastasis-like subgroup was identified accounting for 33.10% of primary melanomas. Tumors identified within the same subgroup were more likely to present with similar clinical features. Kaplan-Meier survival analysis showed that the patients in the ML subgroup have a worse 5-year survival rate compared with patients in the NML subgroup (52.1% vs 76.3%). More importantly, multivariate Cox regression analysis revealed that KAI1-/p27- is an independent prognostic factor in primary melanomas, showing a stronger correlation with patient survival than when used as individual markers. Additionally, we found that melanomas in the ML subgroup exhibited greater thickness compared to the NML subgroup (4.94 vs. 2.66 mm; *P* = 0.001, Table 4). However, we did not detect significant association between KAI1-/p27- signature and other important clinical parameters, such as ulceration (*P* = 0.117, Table 4) and subtype (*P* = 0.83, Table 4). Superficial spreading and nodular subtypes are responsible for most melanoma related-mortality, and the nodular subtype is the most aggressive subtype [49], yet we did not observe significant difference in the distribution of specific subtypes in ML and NML subgroups, which may due to the relatively small case number in each subtype group. Because of a lack of recorded information, we were unable to conduct analyses on other clinicopathological parameters, such as mitotic activity, tumor-infiltrating lymphocyte response, regression, lymph vascular invasion, etc.

Taken together, our results suggest that loss of KAI1 and p27 leads to enhanced metastatic potential for primary melanomas, and that restoring expression or function of KAI1 and p27 can be a potential strategy for melanoma therapy. The KAI1-/p27- signature could potentially identify a distinct subgroup of primary melanoma patients that need to be monitored more closely and treated more aggressively. This study presents a new alternative way of subgroup discrimination which will hopefully facilitate the search for subgroup-specific therapeutic targets and the development of personalized medicine for primary melanoma patients in the metastasis-like subgroup.

## MATERIALS AND METHODS

### Ethics statement

Our study on archival melanoma biopsies was approved by the Clinical Research Ethics Board of the University of British Columbia. The experiments were performed in accordance with the Declaration of Helsinki guidelines.

### Study patients and tissue microarray

The construction of melanoma tissue microarrays (TMAs) and corresponding clinical database have been described previously [30, 33]. The protocol was approved by the Institutional Review Board at the University of British Columbia. Due to core loss, 250 cases could be evaluated for all 7 markers in a training cohort, including 145 primary melanomas and 105 metastatic melanomas. Further, 92 additional cases of primary melanomas were evaluated for both KAI1 and p27. These formed the independent validation cohort used for confirmation analysis of the KAI1-/p27- signature.

### Immunohistochemistry and intensity assessment

The immunohistochemistry staining was performed as described previously [33]. Briefly, Primary rabbit polyclonal anti-BRAF antibody (1:100 dilution; Sigma, St. Louis, MO, USA), rabbit polyclonal anti-Dicer (1:100 dilution; Sigma), mouse monoclonal anti-Fbw7 antibody (1:50 dilution; Abcam, Cambridge, MA), mouse monoclonal anti-KAI1 (1:100 dilution, Novus Biologicals, Littleton, CO, USA), mouse monoclonal anti-MMP2 (1:50 dilution; Biolegend, San Diego, CA, USA), mouse monoclonal anti-p27 (1:100 dilution; Santa Cruz Biotechnology, Santa Cruz, CA, USA), rabbit polyclonal anti-Tip60 (1:50 dilution; Millipore, Temecula, CA, USA) and the biotin-labeled secondary antibody (Dako Diagnostics, Glostrup, Denmark) were used. The technical negative control used for immunohistochemistry included the use of PBS instead of primary antibody, with all other conditions kept the same. Briefly, the staining intensity was scored using the following scale: no staining (0), weak (1), moderate (2), and strong (3). The percentage of positive cells was scored into 4 categories: 1 (0–25%), 2 (26–50%), 3 (51–75%), and 4 (76–100%). The staining intensity and percentage of positive cells were evaluated in a blinded manner by three independent observers (including two dermatologists) simultaneously, and a consensus score was reached for each core. Immunoreactive score (IRS) was used to determine the level of staining by multiplying the scores of staining intensity and the percentage of positive cells. Since cytoplasmic expression of Dicer was correlated with survival in our previous studies, only cytoplasmic Dicer scores were used in this present study. For each biomarker, x-tile software (version 3.6.1) was used to determine the optimized cut-off points, by selecting the maximal χ^2^ values of the log-rank test for survival between two groups [34].

### Statistical analysis

Univariate and multivariate logistic regression analysis was performed to determine the discriminant biomarker between metastatic and primary melanomas. The best two markers were selected by lower *P* values; they were KAI1 and p27 in this study. The Kaplan–Meier survival analysis and log-rank test were used to evaluate the effects of KAI1/-p27- signature on the 5-year melanoma-specific survival. Univariate and multivariate Cox proportional hazards regression analysis were performed to estimate the crude hazard ratios (HRs) or adjusted HRs and their 95% confidence intervals (CI). A *P* value of <0.05 was considered to indicate statistical significance. All statistical analyses were carried out using the SPSS version 16.0 software (SPSS, Chicago, IL, USA).
